# The importance of Ca^2+^-dependent mechanisms for the initiation of the heartbeat

**DOI:** 10.3389/fphys.2015.00080

**Published:** 2015-03-25

**Authors:** Rebecca A. Capel, Derek A. Terrar

**Affiliations:** British Heart Foundation Centre of Research Excellence, Department of Pharmacology, University of OxfordOxford, UK

**Keywords:** sino-atrial node, cardiac, pacemaking, cytosolic calcium, calcium clock, membrane clock

## Abstract

Mechanisms underlying pacemaker activity in the sinus node remain controversial, with some ascribing a dominant role to timing events in the surface membrane (“membrane clock”) and others to uptake and release of calcium from the sarcoplasmic reticulum (SR) (“calcium clock”). Here we discuss recent evidence on mechanisms underlying pacemaker activity with a particular emphasis on the many roles of calcium. There are particular areas of controversy concerning the contribution of calcium spark-like events and the importance of I(f) to spontaneous diastolic depolarisation, though it will be suggested that neither of these is essential for pacemaking. Sodium-calcium exchange (NCX) is most often considered in the context of mediating membrane depolarisation after spark-like events. We present evidence for a broader role of this electrogenic exchanger which need not always depend upon these spark-like events. Short (milliseconds or seconds) and long (minutes) term influences of calcium are discussed including direct regulation of ion channels and NCX, and control of the activity of calcium-dependent enzymes (including CaMKII, AC1, and AC8). The balance between the many contributory factors to pacemaker activity may well alter with experimental and clinical conditions, and potentially redundant mechanisms are desirable to ensure the regular spontaneous heart rate that is essential for life. This review presents evidence that calcium is central to the normal control of pacemaking across a range of temporal scales and seeks to broaden the accepted description of the “calcium clock” to cover these important influences.

## A rudimentary pacemaker

The aim of this review is to discuss the many different roles of Ca^2+^ in regulating pacemaker function in the sino-atrial node (SAN). The major determinants of pacemaker activity remain controversial, as illustrated by reviews from the Lakatta and DiFrancesco groups (Lakatta and DiFrancesco, 2009; DiFrancesco and Noble, 2012a,b; Lakatta and Maltsev, 2012; Maltsev and Lakatta, 2012). Other important reviews have been published in the last 10 years (Dobrzynski et al., 2007; Imtiaz et al., 2007; Wu and Anderson, 2014). A comprehensive review from Mangoni and Nargeot also presents a valuable overview of pacemaker mechanisms, particularly with respect to conclusions drawn from genetic abnormalities and genetic manipulations (Mangoni and Nargeot, 2008). The starting point for the discussion here will be the broadly excellent review by Irisawa et al. (1993) (see also Irisawa (1978) and Noma (1996)) which is a very comprehensive in its discussion of surface membrane currents. Irisawa et al. make little or no inclusion of the possible influence of cytosolic Ca^2+^, particularly that released from the sarcoplasmic reticulum (SR), since little was known on this aspect of pacemaker mechanisms at the time the review was written. In recent years much of the debate concerning the origin of pacemaker activity in the heart has been presented as a choice between two alternative mechanisms, a “membrane clock” in which I(f) activated by hyperpolarization plays the dominant role or a “Ca^2+^ clock” in which the timing of uptake and release of Ca^2+^ by the SR is the major determinant of the cardiac rhythm (DiFrancesco and Noble, 2012a,b; Lakatta and Maltsev, 2012; Maltsev and Lakatta, 2012).

This review seeks to discuss broader aspects of the influence of Ca^2+^ on pacemaker activity than are frequently considered in debates on the relative importance of Ca^2+^ and membrane clocks. The evidence discussed below supports the view that a variety of ionic currents in addition to I(f) can contribute to the timing of the membrane clock, that these events are potentially modulated by intracellular Ca^2+^ in a number of ways and that the relative importance of these pathways might vary under different physiological and clinical conditions. We consider data relating to the role of the Ca^2+^ clock under a range of conditions, and discuss whether such a clock needs to depend solely on spontaneous Ca^2+^ sparks or local calcium releases (LCRs) or whether other rhythmic Ca^2+^-dependent mechanisms should also be taken into account to form a complete picture. It appears that the Ca^2+^ clock could play a fundamentally important role for the timing mechanism of the cardiac pacemaker under particular conditions, but in many circumstances might play a cooperative interacting role with the membrane clock.

Timing mechanisms for different sorts of pacemaker activity have been discussed in many different tissues including oscillations in smooth muscle, interstitial cells, brain and heart (e.g., Berridge and Galione, 1988). Mechanisms include what have been called membrane oscillators and cytosolic Ca^2+^ oscillators in smooth muscle and brain, and ideas concerning a Ca^2+^ clock are not unique to the heart (Imtiaz et al., 2006; McHale et al., 2006; Berridge, 2008; Imtiaz, 2012).

In the heart, a key feature that distinguishes pacemaker tissue from surrounding atrial muscle is the absence of the stabilizing influence of I_K1_. Other important characteristics are the presence of the connexin protein Cx45 (Coppen et al., 1999) and I(f) (Biel et al., 2002) and lack of Cx43 (ten Velde et al., 1995), but the lack of I_K1_ is particularly functionally important for the following reasons. The presence of I_K1_ channels in atrial and ventricular myocytes is responsible for the ~−90 mV resting membrane potential in these cells, dominated by the equilibrium potential for potassium ions in physiological solutions. In the absence of I_K1_ the SAN membrane potential is not forced to “rest” at this potential. In addition, lack of the I_K1_ conducting pathway leads to a greatly increased membrane resistance (reduced conductance) in SAN cells in comparison to atrial and ventricular myocytes and this allows very small ionic currents to exert a profound influence on membrane potential. In this regard, it is also relevant to consider that SAN myocytes exhibit small cell capacitance (of the order of 30–40 pF) in turn requiring only small currents to charge or discharge the membrane capacitance. The significance of the lack of I_K1_ in mammalian SAN pacemaker tissue was first demonstrated in an important paper from Irisawa (Noma et al., 1984) (see also Shibata and Giles, 1985 for similar observations in amphibian pacemaker tissue). The susceptibility to oscillations causing spontaneous activity when I_K1_ is suppressed in ventricular tissue was also shown by Miake et al. (2002).

Although there is no “resting” potential in a pacemaker cell showing continuous electrical activity, an important observation is that when pacemaker activity is arrested (for example by the L-type Ca^2+^ channel blocker nifedipine (Kodama et al., 1997), or by blockers of voltage-gated potassium channels (Lei et al., 2001) the membrane adopts a potential at least for a period of seconds at approximately −30 to −40 mV. A similar potential is adopted when spontaneous activity is stopped by chelation of cytosolic Ca^2+^ with intracellular BAPTA (Capel and Terrar, this issue, and see later). A “resting potential” of −38 mV was also described in rabbit SA node by Noma and Irisawa (1975). Again, a similar potential is recorded in amphibian sinus venosus when spontaneous activity is “arrested” by nifedipine (Bramich et al., 1993). Verheijck et al. (1995) also described a “background current with a reversal potential of −32 mV in rabbit SA node in the presence of nifedipine and E-4031.”

**Figure 1:**
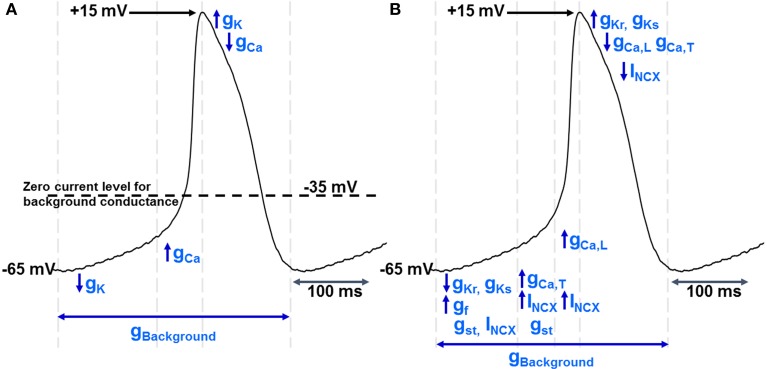
**(A)** shows a simple model of pacemaker function in which there is a “background” current/conductance with a zero current level between −30 and −40 mV. There is extensive evidence for such a pathway, although as discussed in the text the nature of the conductance(s) contributing to this pathway remain poorly understood. However, given the existence of this pathway, pacemaker activity can be maintained by sequential activation of voltage-gated K^+^ and Ca^2+^ ion channels, noting that de-activation of K^+^ conductance (g_K_) after repolarization will be associated with the “diastolic” or pacemaker depolarization as the potential moves toward the zero current level for the “background” conductance. When the membrane potential reaches the threshold for voltage-gated Ca^2+^ channels, activation of these channels (increasing g_Ca_) will lead to the upstroke of the action potential and the depolarization will activate voltage-gated potassium channels to complete the cycle of repetitive activity. The vertical dotted lines show an approximate division of the time period of the pacemaker cycle to represent these phases of channel activation and de-activation. The experimental record that is shown to illustrate these phases was recorded from a guinea-pig SAN myocyte in our laboratory and is similar to records in **Figures 2**, **5** (and also to records in Capel and Terrar in this issue). A formal mathematical representation of these changes in conductance was not constructed, but the analysis is broadly similar to the principles used for the basic mathematical model presented by Hauswirth, Noble, and Tsien in 1968 to account for spontaneous activity in Purkinje fibers (Hauswirth et al., 1968).**(B)** shows a more comprehensive description of the ionic conductances and currents underlying pacemaker activity, including g_CaL_ and g_CaT_ (conductance provided by the two varieties of voltage-gated Ca^2+^ channel in cardiac muscle), g_Ks_ and g_Kr_ (the slowly and rapidly activating voltage-gated K^+^ channels), g_f_ (the conductance associated with channels activated by hyperpolarization and carrying the “funny” current), g_st_ (the sustained inward current channels), and I_NCX_ (the NCX current thought to flow throughout the cardiac cycle as outlined in more detail in **Figure 2**). Again, the vertical dotted lines show an approximate division of the time period of the pacemaker cycle to represent different phases of channel activation and de-activation.

With this “background” conductance as a starting point, a very simple pacemaker can be constructed in which an action potential upstroke carried by calcium ions leads to a depolarisation that then activates a voltage-gated potassium conductance and this in turn brings about repolarisation. Potassium channel de-activation will then lead to a removal of hyperpolarizing influence and allow the membrane to move back toward its “resting” level as a consequence of the influence of the background conductance pathway (Figure 1A). Early modeling work suggested that this mechanism is capable of sustaining spontaneous action potential generation (Hauswirth et al., 1968) and see (Noble et al., 1992).

A more comprehensive model that will be used as a framework for later discussion is shown for comparison in Figure 1B.

Even in a review with an emphasis on the many roles for Ca^2+^, the existence of a background current with a reversal potential in the region of −30 to −40 mV is so fundamentally important for pacemaker mechanisms that it deserves further discussion. It is also conceivable that this poorly understood pathway is itself Ca^2+^-dependent. The first question that arises from the simple model is how the “pseudo resting” level of −30 to −40 mV is determined and what, in turn, is the selectivity of the membrane to different ions at such a “pseudo resting” potential when voltage-gated channels are not active.

## What is background current?

Another way of phrasing the question in the previous paragraph is what is the “background current,” or perhaps better what is the background conductance because little or no net current will flow at the “pseudo resting” potential. Although the evidence presented above in favor of the existence of a background conductance is compelling, there is surprisingly little evidence or agreement on the ion conducting pathways that give rise to this conductance. One approach is to block everything we think we understand and to label what is left as background current e.g., Hagiwara et al. (1992), and this approach leads to the suggestion that background conductance is determined by a balance between potassium conductance (which if dominant would lead to a membrane potential close to the potassium equilibrium potential of perhaps −90 mV) and sodium and/or Ca^2+^ conductances (that if dominant would take the potential to more positive values determined by their equilibrium potentials of ~+60 mV in the case of Na^+^ and more positive than +100 mV in the case of Ca^2+^). This balance could lead to a “pseudo resting” potential of −30 to −40 mV. Chloride ions cannot be excluded and might also contribute [while a contribution of Cl^−^ to “background current” was not detected by Hagiwara et al. (1992), other observations from Seyama (1979) and from Huang (Huang et al., 2009) support a role for Cl^−^].

At rest, single channels with a zero current potential close to the “pseudo resting” level have not been described. Ito et al. (1994) observed activity of I_KACh_ channels in the absence of ACh. These were the only K^+^ channels recorded “at rest” in 105 experiments on SA node cells. These authors concluded that an additional pathway carrying inward current at −50 mV must also exist. These channels could be difficult to find and record from (if they were too scarce, low conductance or too fast and short lived to allow easy detection). One possibility is that a major contributor to background conductance is not an ion channel but NCX in a leak mode as first suggested by Kang and Hilgemann (2004), see later.

Another relevant pathway is the sustained inward current described by Noma and colleagues (Mitsuiye et al., 2000). This pathway cannot contribute to the “pseudo resting” level when nifedipine is used to cause cessation of pacemaker activity, since this drug blocks I(st) in addition to blocking L-type Ca^2+^ channels. However, I(st) is carried by channels that allow passage of monovalent cations and the kinetics of this time and voltage-dependent pathway are sufficiently slow that it could play a role similar to that proposed for the “leak” or background conductance pathway. Under physiological conditions this pathway allows passage of both sodium and potassium ions, with a reversal potential for the current of approximately −20 mV. The single channel conductance of the I(st) pathway has been reported to be 13 pS, and to have an open probability that is regulated by protein kinase A (PKA). In rabbit SAN this pathway gives rise to an approximately 50 pA current at −50 mV in an isolated SAN myocyte (Mitsuiye et al., 1999).

The I(f) current activated by hyperpolarization will be discussed in more detail later, but it is worth mentioning that its kinetics are also slow at potentials close to −60 mV and it could feasibly contribute to conductance over many seconds as well as playing a role in “pacemaker depolarization” during a single cardiac cycle. In this context, Verkerk and Wilders have argued that de-activation of I(f) near the overshoot of the action potential, though fast, is not instantaneous, and significant I(f) can be present in their models at the start of diastolic depolarization, persisting from activation of this pathway during preceding action potentials (Verkerk and Wilders, 2013). In addition, Proenza et al. (2002) suggested that an “instantaneous” current (that could contribute to steady current) might flow via HCN2 channels.

Store Operated Ca^2+^ Channels (STOCCs) could influence heart rate through the generation of a background current that is modulated through beat to beat SR Ca^2+^ content changes (Ju and Allen, 2007) and (Liu et al., 2015). Roles have also been suggested for TRPM4 (Hof et al., 2013) and TRPM7 (Sah et al., 2013).

Other channels that might contribute include BK channels (Lai et al., 2014), SK channels (Chen et al., 2013) and Ca^2+^ activated Cl channels (Verkerk et al., 2002).

## The roles of NCX

### NCX during the later stages of the pacemaker depolarization, perhaps associated with Ca^2+^ sparks/LCRs

**Figure 2:**
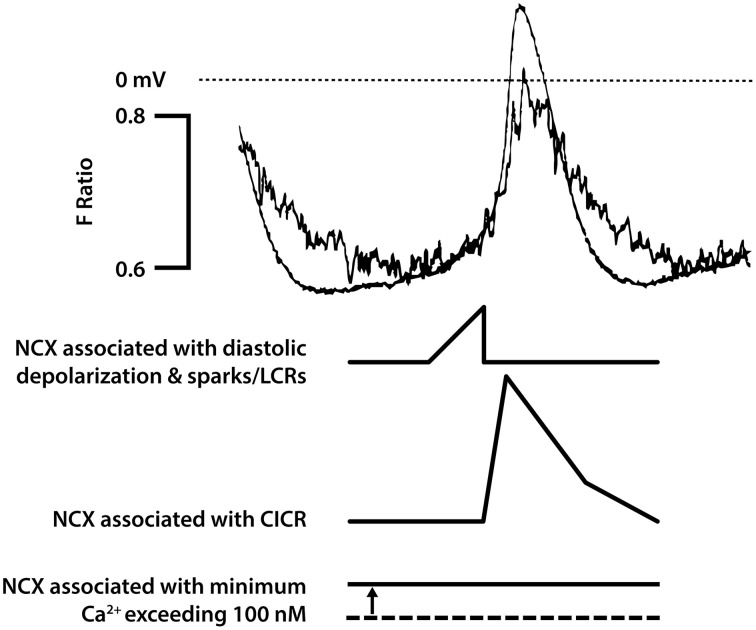
**This figure seeks to show in cartoon form the various contributions of NCX to pacemaker activity [that are not confined just to rises in subsarcolemmal Ca^2+^ associated with spontaneous Ca^2+^ release from the SR described as Ca^2+^ sparks or local calcium release events (LCRs)]**. The magnitude of the NCX currents will broadly follow the magnitude of the Ca^2+^ transient (though if there are sufficient changes in subsarcolemmal Ca^2+^ and Na^+^, these changes will influence the equilibrium potentials for these ions and therefore the driving force for NCX). The time course of the Ca^2+^ transient as reported by the Ca^2+^ probe indo-1 (fluorescence ratio with emission at 405 and 485 nm) is shown overlaid with an action potential in a pacemaker cell in the upper pair of traces. It is recognized that the indo-1 fluorescence ratio (noisy trace) will show a lag as a consequence of the kinetics of Ca^2+^ binding to the probe, and there may be a delay in the decline in Ca^2+^ as a consequence of slow dissociation of Ca^2+^ from the probe (although this delay seems not to be severely limiting since isoprenaline was able to speed the Ca^2+^ signals recorded with this probe, see **Figure 5**). There is a rise in Ca^2+^ that precedes the rapid upstroke of the action potential, and under conditions in which spontaneous Ca^2+^ sparks/LCRs occur these events will contribute to the early phase of NCX. This is followed by CICR triggered by Ca^2+^ entry largely through I_CaL_, and the accompanying large rise in subsarcolemmal Ca^2+^ will be associated with a substantial additional component of NCX. It is thought that the cytosolic and subsarcolemmal Ca^2+^ concentrations remain greater than 100 nM even at the most negative membrane potential between action potentials and this is associated with the third component of NCX represented in the bottom trace, with a dotted line to represent zero NCX current that would be expected to occur at approximately 100 nM Ca^2+^. Although the components of NCX are shown as separate in this diagrammatic representation to emphasize the many roles of NCX, it is recognized that in reality these components run together as a continuum and cannot easily be dissected experimentally. In addition to these three components of electrogenic NCX with a 3 Ca^2+^:1 Na^+^ stoichiometry, there may be an additional “leakage” component (see text). The experimental traces are from Rigg et al. (2000).

There are at least three possible ways in which electrogenic NCX could contribute to pacemaker activity (Figure 2). The most discussed mechanism, often given so much prominence that other possibilities could be overlooked, is the suggestion that there are there are spontaneous events resulting from Ca^2+^ release from the SR [sometimes referred to as Ca^2+^ “sparks” (Huser et al., 2000) or local Ca^2+^ release events (LCRs) (Bogdanov et al., 2001; Vinogradova et al., 2005)] and that the accompanying rises in subsarcolemmal Ca^2+^ cause local depolarizations arising from Ca^2+^ extrusion by electrogenic NCX. These sparks/LCRs can occur in the later stages of the pacemaker depolarization preceding the upstroke of the action potential (Huser et al., 2000; Bogdanov et al., 2001; Vinogradova et al., 2002a, 2005). Although it seems very likely that this is an important component of electrogenic NCX, evidence supports other possibilities listed below that might be equally or under some conditions more important.

### NCX associated with the upstroke of the action potential

In addition to the spontaneous Ca^2+^ release events toward the end of the pacemaker depolarization, there will be the rise in cytosolic Ca^2+^ that is the result of Ca^2+^ entry through Ca^2+^channels during the upstroke of the action potential, and the consequent Ca^2+^-induced Ca^2+^ release (CICR) from the SR. CICR occurs as a global release event (meaning a release that occurs synchronously across all of the SR) arising from ryanodine receptor Ca^2+^ release channels. The global, substantial rise in subsarcolemmal Ca^2+^ accompanying CICR during the upstroke of the action potential will nevertheless be accompanied by a major component of Ca^2+^ extrusion through electrogenic NCX (Figure 2). Even when SR function is suppressed, the substantial influx of Ca^2+^ through L-type channels during the upstroke of the action potential will be accompanied by extrusion of Ca^2+^ and at least some electrogenic extrusion could be fast enough to re-inforce this action potential upstroke. Note that ryanodine has been reported to reduce the maximum rate of rise of the pacemaker action potential, consistent with a contribution of NCX associated with CICR in addition to the charge carried by L-type Ca^2+^ channels (Rigg and Terrar, 1996; Rigg et al., 2000), although other Ca^2+^ dependent mechanisms such as effects of CaMKII on L-type channels could also contribute to this.

### NCX during diastole, including at the most negative potential

The third aspect of possible contribution of NCX to pacemaker activity is a consequence of slower changes in Ca^2+^concentration. It is clear that the level of Ca^2+^ activity in SAN cells between beats does not typically fall to the ~100 nM that is measured in quiescent ventricular or atrial myocytes (e.g., Cannell et al., 1987; Schaub et al., 2006), and has been reported as 225 nM (Sanders et al., 2006). If the level of Ca^2+^ concentration between beats were approximately 200 nM, there would be continuous extrusion of Ca^2+^ even during the intervals between beats. Ca^2+^ balance must be maintained in the steady state, but this does not necessitate discrete entry and extrusion phases. Net Ca^2+^ entry is clearly exhibited during the later stages of pacemaker depolarisation and upstroke of the action potential but Sanders et al suggest that the balancing role of Ca^2+^ extrusion (mainly through NCX) occurs throughout the cycle including the most negative potential between beats. Thus, there could be a maintained depolarizing influence of NCX (Figure 2).

The three aspects of electrogenic 3:1 NCX have been discussed as separate entities above, in order to emphasize their contributions in different parts of the pacemaker cycle, but it is recognized that in reality they represent a continuum of activity that overlap and cannot be easily distinguished. The clear point is that electrogenic 3:1 NCX can play a role throughout the cardiac cycle and will be dependent on the subsarcolemmal Ca^2+^concentration under the control of both local and global events.

The average current through the combined contribution of these three components of NCX is substantial since NCX is thought to be the major method of Ca^2+^ extrusion. Every Ca^2+^ ion that enters the cell via a Ca^2+^ channel (L-type or T-type) adds two charges to the cell interior, while extrusion of each Ca^2+^ by NCX adds one charge (with three sodium ions entering in exchange for each divalent Ca^2+^), and it therefore follows that if the major component of Ca^2+^ extrusion is via NCX then the average depolarizing current through NCX in the steady state throughout the cardiac cycle must be approximately half that through the total Ca^2+^ entry mechanisms.

### NCX acting as a possible sodium leak pathway

A fourth mechanism by which NCX could contribute to pacemaker activity is the leak pathway proposed by Kang and Hilgemann (2004) that was briefly mentioned above in the context of “background current.” The Kang and Hilgemann model was based on extensive experimental evidence using conventional voltage-clamp methods to measure current and voltage across “macro” patches combined with the use of ion sensitive electrodes to give information concerning the sodium and Ca^2+^ gradients close to these membranes. The comprehensive experimental data collected in this way led to the conclusion that the stoichiometry of NCX was 3.2 sodium ions to 1 Ca^2+^, rather than exactly 3:1. The explanation of a 3.2:1 stoichiometry was that there were additional “modes” of NCX. In particular, a mode with slower kinetics that allowed a single external Ca^2+^ together with a single external sodium ion to be exchanged for a single internal Ca^2+^, resulting in electrogenic sodium ion leak without net flux of Ca^2+^, was suggested as a possible contributor to “background current” in pacemaker cells.

The sodium “leak” mode of NCX proposed by Kang and Hilgemann requires the presence of Ca^2+^on both sides of the membrane, but since there is no net movement of Ca^2+^ this mode is not dependent on the driving force for Ca^2+^ entry into the cell. It seems likely, however, that such a pathway could not operate if the cytosolic Ca^2+^ were too low to occupy the internal site on the NCX protein.

### Other aspects of the contribution of NCX for pacemaking

The discussion above focuses on the importance of NCX as a direct contributor to membrane currents. However, in addition to its direct effects as a charge carrier, NCX may have additional functionally important roles in the context of pacemaker mechanisms. In particular these roles include influencing the overall Ca^2+^ balance of the cell (including Ca^2+^ content of the SR), and controlling Ca^2+^ concentrations in cytosolic microdomains within the cell that might influence the behavior/sensitivity of intracellular release channels such as ryanodine receptors (or perhaps IP_3_ receptors see Ju et al., 2011) or Ca^2+^-dependent proteins (see below). Relevant to the present discussion is the thoughtful review by Ottolia et al. (2013) on the importance of NCX in ventricular myocytes. One aspect concerns the balance between NCX and SERCA in determining the amount of Ca^2+^ loaded into the SR which in turn will have an influence on oscillatory Ca^2+^ mechanisms and therefore on Ca^2+^-dependent currents (whether global or local). Although the interplay between NCX and degree of Ca^2+^ loading of the SR has not yet been studied in detail in pacemaker cells, it seems very likely that the mechanisms that operate in ventricular myocytes will have a close parallel in cardiac pacemaker tissue.

A final point that is relevant here is that NCX might be regulated by protein kinases and these could include Ca^2+^-sensitive enzymes (Zhang and Hancox, 2009) as discussed below.

### Is NCX essential for pacemaking?

It is difficult to determine the various contributions of NCX to pacemaker activity by experimental study. There are two problems concerning the use of drugs. The first is selectivity, but even if a drug were available with perfect selectivity for NCX over other cellular targets, the importance of NCX as the major mechanism for Ca^2+^ extrusion under normal conditions means that blockade of NCX would lead to rises in intracellular Ca^2+^concentrations that would have extensive secondary effects. As discussed below, cytosolic Ca^2+^ influences so many ion channels and other aspects of cell function it is difficult to separate primary and secondary effects of NCX suppression. An “ideal” experiment would require instantaneous blockade of NCX so that effects can be observed before substantial secondary effects have time to occur.

Rapid block of NCX can be achieved by reduction of the extracellular solution by replacing some or all of the Na^+^ with Li^+^ (which can pass through most of the ion channels that are permeated by sodium ions but which do not substitute for sodium in NCX). Switch to low sodium does suppress the initiation of spontaneous action potentials, but this suppression can take many beats to establish in toad sinus venosus (Ju and Allen, 1998) and rabbit SA node cells (Bogdanov et al., 2001) when the speed of solution change is modest. When Bogdanov et al caused a rapid but transient solution exchange lasting less than 1 s using a “picospritzer,” they showed that replacement of Na^+^ with Li^+^ could suppress a single action potential (while leaving a slightly depressed Ca^2+^ transient with approximately the same timing as the missing action potential). Sanders et al. (2006) used a rapid switch system that could exchange the solution flowing over individual pacemaker cells in less than 1 s and maintain the flow of this solution. Using this system in guinea-pig SA node cells, rapid switch to low sodium caused immediate cessation of spontaneous activity (both of action potentials and spontaneous Ca^2+^ transients), and this cessation was maintained for the full several seconds of exposure. During this time cytosolic Ca^2+^ as measured by the fluorescent probe fluo-5F fell rather than rose (presumably as a consequence of Ca^2+^ uptake by the SR; see Figure 2 in Sanders et al., 2006).

A possible criticism of sodium replacement experiments is that if the reduction in sodium is very large (e.g., to 14.5 mM or approximately 10%) there may be outward currents through NCX that could complicate interpretation. However, immediate cessation of activity was also seen with smaller reductions of extracellular sodium to 50 mM and even 75 mM (Sanders et al., 2006).

Cessation of SAN myocyte AP firing by low-sodium switch is demonstrably not solely due to suppression of LCR-mediated depolarisation events. Inhibition of Ca^2+^ uptake into the SR using cyclopiazonic acid (CPA), which would be expected after prolonged exposure to abolish SR Ca^2+^ loading, slowed but did not abolish pacemaker activity, and switch to low sodium under these conditions again caused immediate cessation (Sanders et al., 2006). This observation demonstrates the fundamental importance of NCX under these conditions even though the SR is not functional.

Another experimental point arising from the rapid switch experiments is that immediately after the switch back from low sodium to normal sodium the spontaneous rate was restored, but was transiently greater than that observed under control conditions (see Figure 1 in Sanders et al., 2006). This would be consistent with an influence of increased intracellular Ca^2+^ (perhaps an increase in the SR Ca^2+^ load) during the period of exposure to low sodium. In the absence of CPA, when the SR was functional, Sanders et al consistently observed a reduction in cytosolic Ca^2+^ when beating was stopped by the switch to low sodium even though the major mechanism for Ca^2+^ extrusion was inhibited, and this was thought to be accompanied by SR Ca^2+^ uptake (see later).

The observed effects of several drugs that block NCX in causing cessation of pacemaker activity are also consistent with the view that this pathway exerts a profound influence and is probably essential for pacemaking under most conditions (e.g., rapid switch to 5 μM KB-R7943 caused cessation of beating in approximately 20 s, perhaps as a consequence of the time taken to block NCX Sanders et al., 2006), but these observations must be treated with caution because of off target effects of the compounds.

The importance of NCX for pacemaking has also been investigated using genetic approaches to suppress expression of NCX proteins. A knockout of NCX does not survive because it is lethal at the embryonic stage (Cho et al., 2000; Koushik et al., 2001; Reuter et al., 2002). Conditional knockouts in which NCX is selectively suppressed in atrial or SA node tissue have been made. As is the case of drugs, the difficulty of separating direct and indirect effects of NCX suppression applies, and there are compensatory changes that may occur. One recent paper concludes that genetic inhibition (not complete abolition) of NCX1 disables the ability of the SA node to show its normal increase in rate as a “fight or flight” response, but that resting heart rate continues unchanged (Gao et al., 2013). Herrmann et al. (2013) used an inducible SA-node specific Cre transgene to create mice lacking NCX1 in the pacemaker region and showed that ablation of NCX1 was accompanied by a progressive slowing of heart rate and severe arrhythmias. Another recent paper concludes that complete atrial-specific knockout of NCX1 eliminates SA node pacemaker activity (Groenke et al., 2013). In this case the mice were able to survive with the AV node taking over the normal pacemaker function, while the atria appeared to be quiescent. Further, SAN myoyctes isolated when NCX was eliminated also failed to show pacemaker activity.

Taken together the observations above using low sodium, drugs and genetic approaches are consistent with the view that NCX is very important and probably essential for pacemaker mechanisms in the SA node.

## The funny current I(f)

### Is the I(f) current essential for pacemaking?

The “funny” current, I(f) was first described by DiFrancesco and reviewed in DiFrancesco (2010). Similar currents [normally designated I(h)] have been described in neurons showing bursting activity (He et al., 2014). The ion channels carrying the current (HCN channels) are activated by hyperpolarization and the magnitude of the currents in the physiological range is increased when the cyclic nucleotide, cAMP, is bound to the channels [since the activation kinetics are quickened (Wainger et al., 2001) and the activation curve is shifted to less negative potentials, (DiFrancesco and Mangoni, 1994)]. Of the four HCN subtypes, HCN4 is the predominant form in the pacemaker region of the heart, though HCN1 and HCN2 also contribute, and there are species differences between the balance of contributions from different subtypes (Baruscotti and Difrancesco, 2004; Biel et al., 2009; DiFrancesco, 2010).

To answer the question whether or not I(f) is essential for pacemaking, it would be helpful either to block the current pharmacologically, or use the techniques of molecular biology to knock down expression of the channel.

In the case of pharmacological approaches there are a variety of drugs including ZD7288, zetabradine and ivabradine (Baruscotti et al., 2005). Cesium ions have also been used to block the currents (Denyer and Brown, 1990). In all cases the drugs slow but do not completely stop pacemaking. In an early study on SAN myocytes isolated from rabbit SA node 2 mM Cs^+^ was observed to cause close to complete blockade of I(f) without effects on voltage-gated Ca^2+^ and potassium currents. This concentration reduced pacemaker rate by about 30% but did not stop the initiation of action potentials (Denyer and Brown, 1990). The authors concluded that I(f) is not essential, and that there must be an additional “background” current during the pacemaker depolarization (see above discussion). The more recent drugs ZD7288 and ivabradine also slow but do not stop pacemaker activity (BoSmith et al., 1993; Baruscotti et al., 2005). It is, however, difficult to exclude the argument that blockade of I(f) by the drugs is incomplete under the conditions of these experiments, and that only a very small residual current would be necessary for pacemaking. The use of greater concentrations would not be persuasive because of possible non-specific effects such as blockade of L-type Ca^2+^ channels or voltage-gated potassium channels, either or both of which can cause cessation of pacemaker activity.

There has been discussion in the past about whether the I(f) current is sufficiently large to make an important contribution to pacemaking, particularly in the context of conventional experiments in which the cell is held at a constant negative potential (commonly −40 mV) and hyperpolarizing voltage steps are applied. Although large currents are recorded when the cell is hyperpolarized to potentials that are not often experienced in normal physiology (such as −80 or −100 mV), the currents recorded at potentials less negative than −70 mV are small. Convincing evidence for a role of I(f) under conditions that approximate those of normal pacemaker activity is provided by “action potential clamp” experiments (Zaza et al., 1997). The essence of this approach is to record and store the action potential waveform from a single myocyte, and then re-apply this waveform under voltage-clamp conditions. In a single cell the net current under control conditions is zero (since ionic currents charge and discharge the membrane capacitance), but interesting information is provided when blockers are applied to reveal a “difference current” that reflects the particular current pathways that are suppressed by the blocker. Zaza et al. used 2 mM Cs^+^ to block I(f), and concluded that I(f) currents played an important role during the pacemaker depolarization, and that these currents were larger than would have been predicted by conventional voltage-clamp experiments applying only hyperpolarizing pulses, but that other inward currents besides I(f) contribute during this phase.

In the case of genetic experiments, several studies show that suppression of expression of HCN4 proteins also causes a slowing without cessation of activity in the SAN. The most recent uses a conditional knockout system in which HCN4 expression is suppressed only in cardiac tissue of the mouse (Mesirca et al., 2014a). The reduction in spontaneous rate of beating in the SAN reached plateau of about 50% as the I(f) was progressively reduced to very low levels. Interestingly, while the rate reduction in the SAN was reduced by ~50%, there was a cessation of activity in the AV node leading to heart block and death. The apparently greater sensitivity of AV conduction to I(f) suppression seems surprising, but is consistent with the effect of the I(f) blocker ZD7288, which also stops murine AV function at a concentration of 3 μM while reducing spontaneous activity in the SAN by ~50% (Yuill and Hancox, 2002).

In summary, both genetic studies to reduce expression of HCN channels and the effects of selective blockers of I(f) in the mouse show similar effects in that as the severity of action increases to a maximal level the effect on spontaneous rate in the SAN appears to plateau at about a 50% reduction in rate.

There may be other functions of I(f) current in the SAN than determining rate. HCN1 and HCN2 proteins are also present in SAN, and show different kinetics of activation and de-activation compared to HCN4 (Wahl-Schott et al., 2014). Recently knockdown of HCN1 proteins has been shown to influence the stability of pacemaking (and the pacemaker region, Fenske et al., 2013) but not to be essential for pacemaker activity.

### Are I(f) currents influenced by cytosolic Ca^2+^?

Observations from Hagiwara and Irisawa (1989) provide very convincing evidence that that the magnitude of I(f) is influenced by cytosolic Ca^2+^. These authors found that buffering Ca^2+^ at 10^−10^ M caused a substantial (approximately 75%) decrease in current amplitudes, while under the conditions of their experiments 10^−7^ M caused an increase. In experiments in which the cytosol was perfused with different concentrations of Ca^2+^, it appeared that a log(concentration)-response curve could be established showing increases in the amplitude of I(f) over the range of Ca^2+^ concentrations 10^−10^–10^−6^ M with the steepest part of the curve covering the range 10^−8^–10^−7^ M. Although the authors concluded that there was a direct effect of Ca^2+^ on I(f) since the effects of Ca^2+^ were not abolished by the calmodulin inhibitor calmidazolium, it seems likely that the concentration of this compound (10^−6^ M) was not sufficient to cause effective inhibition. This view is supported by the observations of Zaza et al. (1991) who demonstrated that there were no direct effects of Ca^2+^ on I(f) in experiments on inside out patches. Rigg et al. (2003) provided evidence that I(f) is regulated by a Ca^2+^-calmodulin pathway that could at least in part result from a Ca^2+^-calmodulin regulation of adenylyl cyclase, and this possibility is discussed in more detail below.

## Are T-type Ca^2+^ channels essential for pacemaking?

The importance of T-type Ca^2+^ channels for cardiac pacemaker activity has recently been reviewed by Mesirca et al. (2014b). The first evidence for a role for T-type Ca^2+^ channels during pacemaking activity in the SA node was provided by drugs with moderate selectivity, and it was concluded that blockade of these channels caused a slowing but not cessation of rate (Hagiwara et al., 1998). Huser et al. (2000) emphasized a role for T-type channels in triggering Ca^2+^ sparks, though others report that Ca^2+^ sparks can occur without involvement of these channels (Vinogradova et al., 2002a). Genetic studies, particularly concerning Ca_V_3.1 which is the dominant form of T-type Ca^2+^ channel in the adult SA node, show that suppression of this protein causes only modest reduction in heart rate (Mangoni et al., 2006b). It appears from these observations that T-type Ca^2+^ channels can contribute to pacemaker depolarization but make only a modest contribution to pacemaker rate under most conditions.

## Ca^2+^ release from intracellular stores

### Are Ca^2+^ sparks/LCRs necessary for pacemaking?

Huser et al. (2000) first suggested a pacemaker role for Ca^2+^ sparks and associated NCX currents during the later stage of the pacemaker depolarization that precedes the upstroke of the action potential in both subsidiary pacemaker and normal pacemaker myocytes from the cat. Extensive work from the Lakatta laboratory on rabbit SA node cells also shows LCRs as key players in pacemaking (Vinogradova et al., 2002a,b, 2004, 2005, 2006, 2008, 2010; Vinogradova and Lakatta, 2009). Although we have consistently argued for a role of SR Ca^2+^ release in pacemaking (Rigg and Terrar, 1996; Rigg et al., 2000), and this could include an important role for Ca^2+^ sparks/LCRs, it is interesting to note that in our experiments on Ca^2+^ signals in guinea-pig SAN myocytes using both linescan and Nipkow disk techniques (Rakovic et al., 2001; Jackson et al., 2002) we frequently observe cells with apparently normal spontaneous activity that appear to lack detectable Ca^2+^ sparks/LCRs (although the fraction of cells showing this behavior must be regarded as unpublished observations). In addition, it is observed that under most conditions ryanodine slows but does not stop pacemaker activity and as expected this also suppresses LCRs (Vinogradova et al., 2002a). CPA also abolishes LCRs but does not normally cause cessation of pacemaker activity. It therefore appears that Ca^2+^ sparks/LCRs are not essential for pacemaker activity, though when they occur the associated electrogenic Ca^2+^ extrusion via NCX can contribute to the later stages of diastolic depolarization.

### Are synchronized spontaneous Ca^2+^ releases associated with a Ca^2+^ clock essential for pacemaking?

A Ca^2+^ clock mechanism allows for the possibility that there can be generalized Ca^2+^ releases from the SR alternating with periods of Ca^2+^ uptake. Although such a mechanism is normally discussed in the context of LCRs, it is not a requirement for a Ca^2+^ clock mechanisms to have separate local Ca^2+^ events since the release of Ca^2+^ from the SR could be synchronized over all or a large part of the cell when the Ca^2+^ within the SR reaches a critical concentration (leading to a “global” Ca^2+^ release event similar to the CICR described above but arising from events/conditions within the SR). Note that when membrane potential is clamped at a fixed level close to the most negative potential following a period of normal spontaneous beating it is observed that Ca^2+^ transients can occur with a timing and frequency which is close to the frequency and timing that would have been adopted by spontaneous action potentials if firing had not been interrupted (Vinogradova et al., 2004). The spatial dimensions of these spontaneous releases are frequently much greater than those of LCRs, and it seems possible that these large events arise spontaneously as a consequence of properties of the Ca^2+^ release mechanism in the SR. The timing will depend on the kinetics of Ca^2+^ uptake by SERCA and the time it takes for the Ca^2+^ concentration within the SR to reach a critical level for synchronous Ca^2+^ release. The suggestion of the requirement to reach a critical level of Ca^2+^ within the SR is one way in which such a Ca^2+^ clock could operate, though other timing mechanisms are possible for example arising from the time constants of activation/de-activation of critical proteins in the uptake-release sequence controlling Ca^2+^ handling by the SR. The “global” Ca^2+^ clock (whether arising from the time for Ca^2+^ to reach a critical level or from other kinetic properties of the uptake/release process) could provide a driving mechanism for pacemaker activity, but may not be essential for pacemaker activity. The observation that spontaneous pacemaker activity in the form of action potentials is slowed but not stopped (Rigg and Terrar, 1996; Rigg et al., 2000; Bogdanov et al., 2006) when SR function is suppressed with ryanodine and/or CPA shows that synchronized spontaneous Ca^2+^ release from the Ca^2+^ clock is not always essential for pacemaker activity.

**Figure 3:**
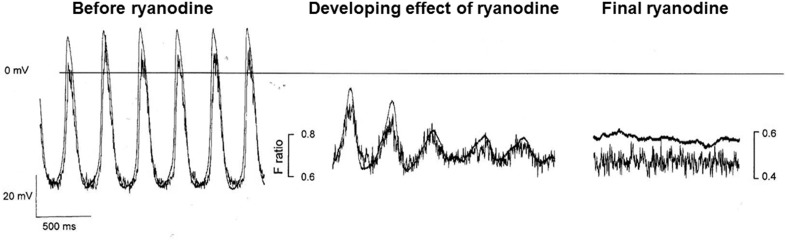
**This figure shows the effect of ryanodine in a pacemaker cell in the presence of the calcium probe indo-1 and isoprenaline**. Although in our experience, ryanodine slows but does not normally stop spontaneous activity, we have observed cessation of activity after ryanodine when calcium probes are present, particularly those with a high Ca^2+^ affinity, and we have attributed this to the additional Ca^2+^ buffering effects of the probe (see text). Despite these limitations, the trace is instructive in that before cessation of activity (which occurs at a potential close to that shown in Figure 1A and described in the text as the zero current level for “background” conductance, see pair of traces on right) subthreshold oscillations occur (middle pair of traces) with a period that is not substantially different from full action potentials at an earlier stage of ryanodine effect. These membrane potential fluctuations are mirrored by corresponding fluctuations in the Ca^2+^ fluorescence signal. The experimental traces are from Rigg et al. (2000).

Although under most conditions, ryanodine does not stop spontaneous activity, we have sometimes observed cessation when we have used Ca^2+^ probes with high Ca^2+^ affinity, such as indo-1 (perhaps associated with the additional Ca^2+^ buffering properties of such probes, Rigg et al., 2000). Figure 6 from Rigg et al. is reproduced here showing the progressive development of the effect of ryanodine with a high concentration of isoprenaline present (Figure 3). After 4 min exposure to ryanodine, there is oscillating electrical activity that is so small that it must be subthreshold for the normal action potential mechanism but which nevertheless shows a repeating activity with the same period as the underlying Ca^2+^ signal (and is slightly slowed compared with spontaneous action potentials recorded in the same cell). The observed oscillations recorded during the development of the effect are best explained by a Ca^2+^ clock that is driving electrical activity presumably through NCX.

There is a very impressive body of evidence showing that the timing of the Ca^2+^ clock (as judged from the frequency of spontaneous Ca^2+^ events when the membrane potential is held at a constant membrane potential) is correlated with the timing of the action potentials on the surface membrane recorded when spontaneous electrical activity is allowed to occur (Vinogradova et al., 2002a,b, 2004, 2005, 2006, 2008, 2010; Bogdanov et al., 2006; Vinogradova and Lakatta, 2009). This has been examined over a broad range of frequencies when the rate is either increased by adrenoceptor agonists or decreased with muscarinic agonists, and the correlation between membrane and Ca^2+^ oscillations remains very strong throughout the range of frequencies studied. There is a parallel correlation between timing of the Ca^2+^ clock and the degree of phosphorylation and dephosphorylation of functionally important proteins such as L-type Ca^2+^ channels and phospholamban (Vinogradova et al., 2006). Although the correlation between the timing of the Ca^2+^ clock and the frequency of spontaneous action potentials is impressive, this does not establish that the relationship is always causal.

It could be argued that the timing of release and uptake of Ca^2+^ by the SR needs to keep pace with events in the surface membrane in order for a pacemaker mechanism driven primarily by the surface membrane to be maintained in an efficient way, without any disturbance that might otherwise arise from competition with these SR-dependent mechanisms. In other words, the primary mechanism driving the timing of the SA node pacemaker could still depend on the kinetics of activation and deactivation of membrane proteins making up the membrane clock. However, a coupled clock mechanism (Imtiaz et al., 2006, 2007, 2010; Yaniv et al., 2011, 2013; Imtiaz, 2012; Maltsev et al., 2013, 2014) in which both membrane and Ca^2+^ clocks exert mutually interdependent influences also seems plausible, and perhaps more likely under many circumstances because the system will function most efficiently when the surface membrane and “Ca^2+^ clock” mechanisms are not competing against one another. The two clocks would normally be synchronized, and this may be the optimal configuration. When the Ca^2+^ clock does play a role, there are a variety of ways that cytosolic Ca^2+^ will influence the periodicity of the clock, including influencing the rate of uptake of Ca^2+^ by SERCA and in turn the time taken to reach a critical level within the SR. Other longer term mechanisms for Ca^2+^ to influence the clock by actions on Ca^2+^-dependent enzymes are considered below. However, it is clear from the discussion above, and particularly from the observation that pacemaker activity can occur without a functional SR, that the Ca^2+^ clock is not essential for spontaneous pacemaker activity supported by a membrane clock.

### Do abnormalities in Ca^2+^ handling by the SR influence pacemaker rate?

If the Ca^2+^ handling properties of the SR can provide an important timing mechanism that under some conditions drives the generation of action potentials in the SA node, it might be speculated that defects in the function of the SR could lead to rhythm abnormalities.

An abnormality concerning ryanodine receptor Ca^2+^ release channels in the SR membrane supports such a possibility (Neco et al., 2012). This was studied in mice carrying the catecholaminergic polymorphic ventricular tachycardia–linked mutation of ryanodine receptor (RyR2^R4496C^). The SAN region was studied using confocal microscopy and the Ca^2+^ probe fluo-4 at room temperature. The SAN from RyR2^R4496C^ mice showed slowed pacemaker activity and reduced response to catecholamines as compared to myocytes from WT. There were also frequent pauses in the generation of spontaneous action potentials, while the overall frequency of Ca^2+^ sparks/LCRs was increased. Experiments were also carried in SAN preparations loaded with rhod-2 to measure Ca^2+^ and di-4-ANEPPS to measure membrane potential. Pauses interspersed with trains of spontaneous events were observed for both the Ca^2+^ and voltage signals. The pauses were associated with unusually high cytosolic Ca^2+^ concentrations, and it was suggested that the pauses may have resulted from inactivation of the L-type Ca^2+^ channels.

A recent paper from the Federov laboratory (Glukhov et al., 2015) also shows that pacemaker activity is altered when Ca^2+^ handling is modified in mice lacking the SR protein calsequestrin (*Casq2^−/−^*). These mice showed bradycardia and beat to beat heart rate variability. SAN myocytes isolated from *Casq2^−/−^* mice showed an unstable rate with frequent pauses between “trains” of action potentials and the abnormal behavior was associated with abnormal Ca^2+^ release from the SR. In particular the pauses were accompanied by elevated levels of diastolic Ca^2+^ concentration (Glukhov et al., 2015). The effects of calsequestrin lack are complex reflecting the central role of calsequestrin not just as a Ca^2+^ buffer, but as a protein that together with junction and triadin, appears to regulate Ca^2+^ release via ryanodine receptor releases channels in the SR membrane. The responses to isoprenaline were also abnormal for both the chronotropic effect in whole animals and for the change in rate in isolated SAN myocytes. The authors proposed that “the observed pauses in between rhythmic Ca^2+^ transients in *Casq2^−/−^* SAN cells are caused by excessive diastolic SR Ca^2+^ release in turn resulting in (i) inhibition of the SAN upstroke I_CaL_ current from partially inactivated L-type Ca^2+^ channels, and/or (ii) decrease in the SR Ca^2+^ content below a threshold level required for the generation of spontaneous SR Ca^2+^ releases.”

Another important point in this context is a possible parallel between a Ca^2+^ clock in pacemaker tissue and ventricular arrhythmias in which Ca^2+^ release from the SR is proposed to occur when the Ca^2+^ concentration within the SR reaches a specific level detected by a “calcium sensing gate” of the ryanodine receptor (RyR2). This “store overload-induced Ca^2+^ release” (SOICR) is modified in ventricular myocytes when a point mutation is introduced in the proposed calcium sensing gate (Chen et al., 2014) [and see (Sitsapesan and Williams, 1994, 1997) for evidence concerning the regulation of RyR2 gating by SR luminal Ca^2+^]. Carvedilol and a structural analog which lacks ability to block β-adrenoceptors both block SOICR (Zhou et al., 2011; Maruyama et al., 2013). The possible similarity between a pacemaker Ca^2+^ clock and SOICR has received support from another paper involving the same authors in experiments showing that the carvedilol analog and ryanodine both reduced spontaneous rate and also reduced the positive chronotropic effect of isoprenaline (Shinohara et al., 2014).

Taken together, the observations above show very clearly that pacemaker activity is disrupted when Ca^2+^ handling by the SR is abnormal and demonstrate that at least under some conditions the SR can play a central role.

## Ca^2+^-dependent enzymes

Most of the pacemaker mechanisms described above play a role over times of seconds or less. However, there are important effects of Ca^2+^ that may take much longer to take effect, including activation of a variety of Ca^2+^ dependent enzymes.

### CaMKII

An important Ca^2+^-dependent enzyme for which there is very convincing evidence concerning a role during pacemaking is CaMKII. Evidence using selective drugs supports an important role for CaMKII in maintaining and regulating pacemaker activity in the rabbit SA node (Vinogradova et al., 2000). It was emphasized that CaMKII has properties to function as a “frequency detector” and is therefore well suited to the regulation of pacemaker activity. The CaMKII inhibitor KN93 (but not the inactive analog KN92) substantially slowed or even stopped pacemaker activity. Similar observations were made using the peptide inhibitor AIP. The protein targets for CaMKII activated by Ca^2+^ were shown to include L-type Ca^2+^ channels. Phospholamban regulating the uptake of Ca^2+^ into the SR is another important target. Observations concerning the effects of KN93, KN92, and autocamtide inhibitor peptide (AIP) on spontaneous action potentials in guinea pig SAN myocytes (Xie et al., 2015) were broadly similar to those in the rabbit.

Genetic approaches also provide evidence for the influence of CaMKII on pacemaking at least under stress conditions. One interesting approach is to overexpress an inhibitor peptide for CaMKII (Vinogradova et al., 2000; Chen et al., 2009; Wu et al., 2009; Gao et al., 2011). A “control” is also provided by overexpression of a modified inhibitor protein that lacks ability to suppress CaMKII. In these experiments overexpression of the peptide inhibitor for CaMKII in mouse had little or no effect on resting heart rate (for experiments *in vivo*, in isolated Langendorff perfused hearts and in isolated SA node myocytes), but suppression of CaMKII activity by the peptide did cause a substantial reduction of the positive chronotropic response to isoprenaline. The responses that were reduced by inhibition of CaMKII included the amount of Ca^2+^ loaded in to the SR, the frequency of Ca^2+^ sparks/LCRs and the slope of the diastolic potential. However, interestingly the enhancement of L-type Ca^2+^ current amplitude by isoprenaline was maintained when CaMKII activity was suppressed by the inhibitor peptide. Another mouse model also showed little or no effect of CaMKII knockout on resting rate (Wu et al., 2009) but the positive chronotropic response to isoprenaline was again reduced (and indeed even a slowing of heart rate was seen in the CaMKII KO mice exposed to isoprenaline). These observations support a role for CaMKII in making a major contribution to the “fight or flight” mechanisms in which pacemaker activity is speeded up by adrenoceptor mediated cell signaling. It is clear that CaMKII is a Ca^2+^ stimulated enzyme that can influence pacemaker activity, even if the role of this enzyme under resting conditions remains to be fully established. The importance of CaMKII for pacemaker activity in the SA node has been recently reviewed (Wu and Anderson, 2014).

### Adenylyl cyclases including AC1 and AC8

There are important differences between ventricular myocytes and those in the atria and SA node in terms of the subtypes of adenylyl cyclases, and of the basal activity of these enzymes. In particular it has been shown that actions of ACh at rest in the atria are consistent with inhibition of ongoing basal activity of ACs, while in the ventricle inhibitory actions of ACh are readily detected when adenylyl cyclases are stimulated by aderenoceptor activation but not in the absence of this stimulation (Fischmeister and Hartzell, 1986; Hescheler et al., 1986; Fischmeister and Shrier, 1989; Petit-Jacques et al., 1993). One possibility in the context of the importance of Ca^2+^ for pacemaking mechanisms is the possibility that Ca^2+^-sensitive adenylyl cyclases are preferentially expressed in the atria and SA node as compared with the ventricle. Evidence that I(f) is regulated by a Ca^2+^-calmodulin dependent mechanism that is not CaMKII was presented by Rigg et al. (2003) who also speculated that Ca^2+^ regulated adenylyl cyclase could provide such a mechanism. Application of the Ca^2+^ chelator BAPTA reduced I(f) as did calmodulin inhibitors including W7. Although W7 is known to have non-specific effects (Chatelier et al., 2005), these seem unlikely to be solely responsible for the observations since effects of BAPTA on I(f) were no longer seen in the presence of W7. More direct evidence for the presence of Ca^2+^-stimulated adenylyl cyclases was presented by Mattick et al. (2007). The Ca^2+^-stimulated adenylyl cyclase AC1 was shown to be expressed in the SAN by RT-PCR methods to detect mRNA as well as by immunoblotting with a specific antibody. Confocal microscopy also showed staining with the specific antibody in SAN myocytes. In addition to AC1, another Ca^2+^-stimulated adenylyl cyclase AC8, was detected by immunocytochemistry in the SAN myocytes. Functional evidence using BAPTA and selective inhibitors was also presented to show the importance of this pathway for regulating I(f) although it was also pointed out that such a pathway might well have additional effects via PKA and phosphorylation of additional protein targets. Younes et al. (2008) provided further evidence using RT-PCR for the presence of Ca^2+^-stimulated AC1 and AC8, in addition to AC types 2, 5, and 6 in SAN myocytes. These experiments also demonstrated the functional importance of the high basal activity of adenylyl cyclase in SA node myocytes for the regulation of a variety of proteins phosphorylated by PKA.

In addition, Kryukova et al. (2012) carried out experiments in which AC1 or AC6 were co-expressed with HCN2 in neonatal rat ventricular myocytes that lack normally AC1, and concluded from their observations that AC1 could play an important Ca^2+^-dependent role in regulating HCN2.

The importance of the Ca^2+^ stimulated adenylyl cyclases (AC1 and/or AC8) for the regulation of L-type Ca^2+^ currents has recently been demonstrated in atrial myocytes, and the observations may have relevance to SAN function (Collins and Terrar, 2012). In these experiments on atrial myocytes it was observed that buffering of cytosolic Ca^2+^ with BAPTA reduced Ca^2+^ current amplitude, as did inhibition of Ca^2+^ release from the SR using ryanodine and thapsigargin. Interestingly, the effects of BAPTA were prevented by inclusion of cAMP in the patch pipette. The observations are therefore consistent with a role for Ca^2+^ (including those released from the SR) in stimulating adenylyl cyclases and regulating the magnitude of L-type Ca^2+^ currents. It is speculated that similar mechanisms may operate in the SA node.

A variety of other Ca^2+^ dependent enzymes have the potential to influence pacemaker activity but have not yet been studied in detail. These include phophodiesterases, phosphatases and P21-activated kinase (PAK) enzymes that regulate phosphatases (Ke et al., 2007; Chen et al., 2014; Wang et al., 2014). There may also be effects of Ca^2+^ on NO synthase(s) in SAN myocytes since evidence supports actions of NO donors on pacemaker function (e.g., Han et al., 1995; Yoo et al., 1998; Musialek et al., 2000).

## Ca^2+^ regulation of K^+^ channels

The importance of voltage-gated potassium channels, not only for repolarization of the action potential but also as a contributor to pacemaker depolarization as a consequence of time-dependent de-activation, was emphasized at the start of this review (and see Clark et al., 2004). Evidence that the contribution of potassium channels is subject to regulation by cytosolic Ca^2+^ was provided by Zaza et al. (1997) using the “action potential” clamp methods in rabbit SA node mentioned above in relation to I(f). Zaza et al. (1997) concluded that Ca^2+^ influx during the pacemaker cycle also increases a potassium conductance. This potassium conductance could arise from a single channel component or multiple components.

Xie et al. (2015) have recently presented evidence that the slow component of the delayed rectifier current (I_Ks_) is regulated by a Ca^2+^-dependent process thought to be CaMKII in guinea-pig SA node. This current was found to be decreased when the cytosolic Ca^2+^ concentration was reduced from 10^−7^ to 10^−10^ M (buffered by EGTA in a ruptured patch conditions). When the cytosolic Ca^2+^ concentration was maintained at 10^−7^ M, addition of calmodulin via the patch pipette increased the current. Inhibitors of CaMKII (autocamtide-2 inhibitor peptide and the less selective KN-93) substantially reduced the current, while in the presence of KN-93 there was no effect of calmodulin addition. Taken together these observations provide convincing evidence that I_Ks_ is modulated by cytosolic Ca^2+^ and that CaMKII plays an important role in this modulation.

Might other channel components be involved? The action potential clamp experiments of Zaza et al. mentioned above were carried out in rabbit SA node, and in this species the rapidly activating I_Kr_ is thought to provide the major voltage-gated potassium current pathway (Verheijck et al., 1995; Lei and Brown, 1996). Although under the conditions of the action potential clamp experiments, a Ca^2+^ dependence of I_Kr_ was not detected, applications of nifedipine to block Ca^2+^ entry may have been too brief (less than 10s in Figure 2 of Zaza et al., 1997 while the full effect of Ca^2+^ on an enzyme pathway might take a period of minutes to develop). More evidence against Ca^2+^ regulation of I_Kr_ is provided by Wu and Anderson (2014). However, it has been shown that I_Kr_ in guinea pig ventricular cells can be influenced by cytosolic Ca^2+^ (Heath and Terrar, 2000) in a complex pathway perhaps involving PKC that depends on Ca^2+^ entry via L-type Ca^2+^ channels leading changes in the amplitude and rectification/inactivation of the channel. It therefore seems premature to exclude an effect of Ca^2+^ on I_Kr_ in SA node at this stage.

An interesting additional point in the debate on Ca^2+^ regulation of potassium conductance in the SA node concerns the possible contribution of the Ca^2+^ activated potassium channel BK (Imlach et al., 2010; Lai et al., 2014) and SK (Chen et al., 2013).

## Are there effects of the SR that are independent of the Ca^2+^ clock mechanism?

As was mentioned above, membrane and Ca^2+^ clocks might normally be synchronized, and consequently CICR could occur at (or very close to) a time when “global” spontaneous Ca^2+^ release would also have occurred. However, it is also possible that CICR can, and perhaps often does, occur without the accompaniment of spontaneous Ca^2+^ release. In this case Ca^2+^ released by CICR from the SR would be expected to drive a significant component of NCX that is independent of the Ca^2+^ clock.

Ca^2+^ released from the SR by CICR that is independent of the Ca^2+^ clock could also drive Ca^2+^-dependent enzymes. These include CaMKII, AC1 and AC8 as described above. A role for SR-released Ca^2+^ (sensitive to ryanodine and thapsigargin) leading to increased L-type Ca^2+^ currents by a mechanism involving Ca^2+^-stimulated adenylyl cyclases was recently demonstrated in atrial myocytes (Collins and Terrar, 2012), and it seems very likely that a similar mechanism operates to control pacemaker activity in SAN myocytes. Such a Ca^2+^-induced stimulation of adenylyl cyclases that is at least partly dependent on Ca^2+^ released from the SR by CICR could also contribute to stimulation of I(f) (by elevation of cAMP) and other ion channels such as I(st) and voltage-gated potassium channels (regulated by PKA).

## What are the functions of L-type Ca^2+^ channels associated with SAN action potentials?

Voltage-gated L-type Ca^2+^ channels are a major component of the membrane oscillator providing explosive positive feedback for the upstroke of the action potential which in turn initiates repolarization as a consequence of activation of voltage-gated K^+^ channels. The positive feedback for activation of L-type Ca^2+^ channels underlies the fast upstroke and rapid spread of the action potential that synchronizes Ca^2+^ entry and CICR across the cell. The Ca^2+^ entry via L-type Ca^2+^ channels is a major determinant of the degree of Ca^2+^ loading of the SR. The action potential signal is essential for conduction of the pacemaker activity to neighboring myocytes. In addition, it appears that current through Ca_V_1.3 channels can contribute to the late stage of diastolic depolarization (Zhang et al., 2002; Mangoni et al., 2003, 2006a; Christel et al., 2012). It is also important to note that Ca^2+^ ions entering the cell via L-type Ca^2+^ channels, at any stage during the cardiac cycle but particularly during the rapid upstroke, will drive NCX and cause the extensive secondary effects discussed elsewhere in this review, including enhancement of the activities of PKA and CaMKII (see later).

## Application of Ca^2+^ chelators to reduce cytosolic Ca^2+^ to 10 nM or less

At the present time, it is probably only possible to investigate the effects of Ca^2+^ chelators in single cells, since in multicellular preparations it is difficult to be sure that uptake of Ca^2+^ chelator is complete. It is recognized that isolated myocytes might behave differently from those in the intact myocardium for a variety of reasons including different mechanical influences, lack of paracrine influences from neighboring cells and lack of electrical connections to adjacent myocytes.

In the case of isolated myocytes, Zaza et al. (1997) reported that “under buffering of intracellular Ca^2+^ (ruptured patch, 10 mM intracellular EGTA), spontaneous activity became unstable and ceased in the majority of cells several minutes after achieving the whole-cell configuration.” Another approach to apply the Ca^2+^ chelator is to expose the cells to the acetoxymethyl (AM) ester of the Ca^2+^ chelator applied in the extracellular solution. The membrane permeant ester enters the cell and the active chelator is liberated following the action of esterases in the cytosol. Application of EGTA-AM or BAPTA-AM in this way causes cessation of beating (e.g., Sanders et al., 2006), although it is possible that unspecific effects such as blockade of I_*Kr*_could contribute to these actions (Tang et al., 2007). Loading of SAN myocytes with AM esters of Ca^2+^ probes such as indo-1 can also lead to cessation of activity, and it was suggested that this could arise from Ca^2+^ chelation by the probe (Rigg et al., 2000). One important exception concerning effects of BAPTA on pacemaker activity is the paper by Himeno et al. (2011) showing that pacemaker activity in the form of action potentials can continue while contraction of the cells is suppressed by application of cytosolic BAPTA. However, these authors also noted that “action potential generation became irregular or stopped completely ~5 min after the application of BAPTA in our experiments.” What is the significance of the observations very soon after breakthrough? In support of their contention that buffering is effective even 20 s after breakthrough the authors show a prolongation of action potentials (thought to arise from slowing of inactivation of Ca^2+^ currents) that would be consistent with the chelating effects of BAPTA. Maltsev et al. (Maltsev et al., 2011; Yaniv et al., 2011) have challenged the significance of these observations with the suggestion that breakthrough from perforated patch to “whole” cell patch weakens the “seal” around the electrode noting that such a weakened seal could allow a “leakage” current that could substitute for a physiological current that is blocked by BAPTA. In other words they maintain that spontaneous activity can continue with little change after chelation of BAPTA since leakage current via a weakened “seal” around the patch pipette can replace the depolarizing influence that would normally be supplied by NCX (or any other Ca^2+^-sensitive “background” current that occurs when Ca^2+^ is maintained at a physiological level). In support of this suggestion they note that in Figure 5 of Himeno et al. there is an upward deflection on the contraction trace following breakthrough that would be consistent with a small developing contraction associated with entry of Ca^2+^ from the extracellular solution via the leaky seal into the cytosol. Contribution of leakage current around a seal in sustaining pacemaker activity has been considered by other authors, and can make an important contribution under some conditions (Denyer and Brown, 1990), though these authors concluded from observations comparing the effects of Cs^+^ under perforated patch conditions and in unpatched cells that the effects of the leakage current can be negligibly small if the seal is sufficiently robust.

This point is addressed in another paper in this issue of Frontiers in Physiology (Capel and Terrar, 2015). It is reported that under the conditions of these experiments, rupture of the membrane beneath a patch pipette to apply BAPTA to the cytosol can cause rapid cessation of activity. When conventional whole-cell patch recording was used with 10 mM BAPTA in the patch pipette solution, this cessation of spontaneous activity was almost immediate (less than 10 s). When the same rupture of the membrane beneath the pipette was made without BAPTA present the spontaneous activity appeared not to be reduced throughout the period of recording (approximately 3 min). When action potentials were first recorded with permeabilised patch (with amphotericin in the pipette to approximate the conditions of Himeno et al.) followed by rupture of the patch membrane, the disruption of spontaneous activity after cytosolic application of 10 mM BAPTA was still clearly observed but slower to develop (perhaps because of effects of amphotericin or its solvent). The speed of disruption depended on the concentration of BAPTA, progressively decreasing over the range 10, 1, and 0.1 mM BAPTA.

**Figure 4:**
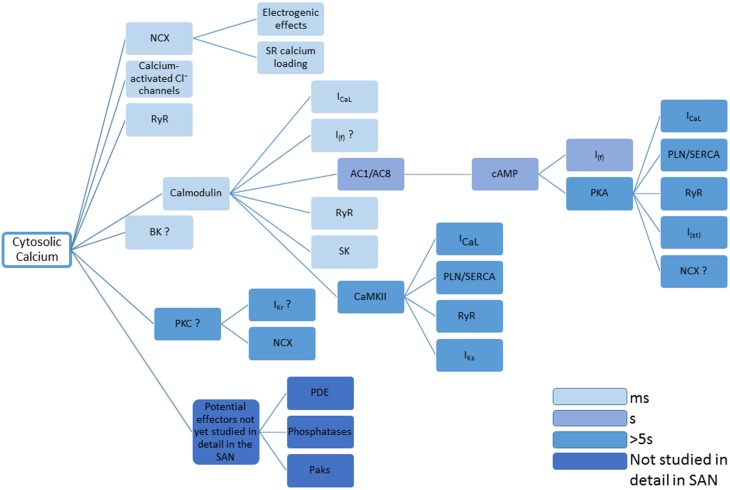
**This figure summarizes the many disparate roles for Ca^2+^ in controlling the activity of proteins that determine pacemaker activity**. Question marks represent cases in which there is currently limited evidence, and references to support the actions shown can be found in the main text. The entries are color coded to give an approximate indication concerning time taken for changes in cytosolic Ca^2+^ to take effect. The figure concentrates on the effects of cytosolic Ca^2+^, though it will be the case that Ca^2+^ within the SR will have additional effects, for example through calsequestrin or RyR2 (either via a possible Ca^2+^ sensor within the RyR structure, or through inactivation of RyRs that might be influenced by SR Ca^2+^). Also omitted are the possible roles of NO produced via Ca^2+^-stimulated nNOS and eNOS. Small molecules controlling Ca^2+^ signaling (e.g., IP_3_, see Ju et al., 2011) are also not included.

The many effects of Ca^2+^ on the mechanisms underlying pacemaker activity and the time windows within which they are thought to act are summarized in Figure 4.

## Catecholamines and sympathetic nerve stimulation

Evidence concerning the mechanisms underlying the positive chronotropic actions of isoprenaline have been recently discussed with particular reference to computer models of pacemaker activity (Zhang et al., 2012). In the context of the review presented here, the focus will be on the importance of the many Ca^2+^-dependent mechanisms that are enhanced during β-adrenoceptor function. The importance of the SR in contributing to the positive chronotropic actions of isoprenaline was first demonstrated by Rigg et al. (2000) in experiments on both intact SA node and isolated SAN myocytes. After exposure to ryanodine, the log(concentration)-response curve for the effects of isoprenaline on intact node was depressed with a reduced slope and maximum effect.

Often effects of isoprenaline (isoproterenol) acting on β-adrenoceptors and of sympathetic nerve stimulation are treated as equivalent, although some have argued that this is over-simplistic (Bramich et al., 1993; Choate et al., 1993; Bramich and Cousins, 1999). There seems justification in this point of view, though a detailed discussion is beyond the scope of this review except to point out that the effects of nerve released noradrenaline on α and β adrenoceptors, as well as the effects of co-transmitters such as ATP and peptides (e.g., Herring, 2014) might have additional effects on Ca^2+^-dependent pathways.

A major effect of β-adrenoceptor stimulation by isoprenaline is to increase Ca^2+^ currents. Many authors agree that this effect is a major factor in the positive chronotropic effect (Brown et al., 1975; Zhang et al., 2012), and an interesting possibility is that there may be important effects of β-adrenoceptor stimulation to increase current through Ca_V_1.3 channels that appear to contribute to the later stages of diastolic depolarization (Zhang et al., 2002; Mangoni et al., 2003, 2006a). However, as mentioned above, the effects of β-adrenoceptor stimulation cannot result from the direct effects on the upstroke of the action potential alone as a consequence of increased L-type Ca^2+^ currents. This is because the upstroke makes up only about 10% or less of the cardiac cycle, and so even if the upstroke became infinitely fast (in other words effectively vertical), the pacemaker period would only be reduced to 90% of the value before the increase in Ca^2+^ current (or a rate increase of 1/0.9 giving close to a 10% increase in rate). The increase in upstroke velocity would still be functionally useful in increasing the rate of conduction of the pacemaker message in the form of the action potential to surrounding tissue. However, in terms of the rate change it seems very likely that an important aspect of the β-adrenoceptor mediated increase in Ca^2+^ current is to increase entry of Ca^2+^ which in turn influences many of the Ca^2+^-stimulated processes that have been described above.

The most obvious of these is NCX. Broadly if Ca^2+^ entry were to double, the cell would need, in the steady state, to remove twice as much Ca^2+^ and if the major mechanism for achieving this were NCX in its normal 3:1 mode then NCX would also double (and the total charge moved by NCX would remain approximately half that of the total Ca^2+^ entry through channels). Although this seems very likely to be the case, one additional point concerns another effect of β-adrenoceptor stimulation via cAMP and PKA. This is phosphorylation of phospholamban which will lead to increased Ca^2+^ uptake by SERCA into the SR. This might lead to a greater fraction of the Ca^2+^ to be taken up by the SR (or at least a greater rate of uptake into the SR depending on how quickly the Ca^2+^ is released), but in the steady state increased Ca^2+^ entry through the surface membrane must be accompanied by increased extrusion. In terms of the Ca^2+^ clock, the kinetics of Ca^2+^ uptake are expected to have a profound effect, and this might be one factor underlying the reduced effect of isoprenaline after ryanodine (Rigg et al., 2000), though other factors might also contribute as discussed below.

**Figure 5:**
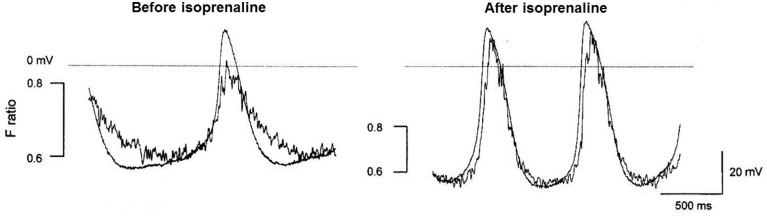
**This figure shows the Ca^2+^ transient reported by indo-1 (fluorescence ratio with emission at 405 and 485 nm) accompanying spontaneous action potentials in the same cell in the presence and absence of isoprenaline**. It can be seen that the increase in rate of occurrence of spontaneous action potentials caused by isoprenaline was accompanied by an increased amplitude of the Ca^2+^ transient, an increased rate of rise of the Ca^2+^ transient and an increased rate of decay of the transient. The same limitations outlined in Figure 2 apply here concerning a possible lag in the response of the Ca^2+^ probe to a rise in Ca^2+^ and possible slowing of the time course (though clearly any such delays do not prevent the detection of the speedier decay of the Ca^2+^ transient in the presence of isoprenaline). The experimental traces are from Rigg et al. (2000).

Consistent with the above suggestions of increased Ca^2+^ transients in single SAN myocytes, Rigg et al. (2000) recorded substantial increases in the amplitude as well as decay of Ca^2+^ transients recorded with the Ca^2+^ probe indo-1 in the presence of isoprenaline (see Figure 5). The effects of autonomic transmitters on Ca^2+^ transients and action potentials in single SAN myocytes have also been reported by Vinogradova et al. (2002a) and van Borren et al. (2010).

The additional Ca^2+^ both entering through L-type channels, and released from the SR (in individual releases and even more as a time average over many beats) could drive many Ca^2+^-dependent processes, such as Ca^2+^-stimulated enzymes and these are expected to contribute to the positive chronotropic effect. The Ca^2+^-stimulated enzymes include CaMKII as discussed by Wu and Anderson (2014), as well as the Ca^2+^-stimulated adenylyl cyclases, AC1 and AC8. As discussed above, Ca^2+^-activation of these enzymes is expected to increase I(f), I(st), I_Ks_, I_Kr_ and possibly NCX, as well as I_CaL_.

## ACh and parasympathetic nerve stimulation

Effects of acetylcholine include both inhibition of adenylyl cyclase and opening of I_KACh_ channels. The effects on I _*KACh*_ channels are blocked by tertiapin Q. During parasympathetic nerve stimulation there is a rapid phase of slowing that is sensitive to tertiapin Q and therefore I_KACh_ (Bolter and Turner, 2009). The additional effects of ACh to inhibit adenylyl cyclase are in many respects the opposite of β-adrenoceptor stimulation discussed above. Consequently the inhibition of L-type Ca^2+^ current will again be very important, not so much for direct effect on the rate of rise of the action potential, but for the consequences of reduced Ca^2+^ entry on the many Ca^2+^-dependent processes discussed above. In the context of Ca^2+^-dependent mechanisms and in particular the Ca^2+^ clock Lyashkov et al. (2009) show that ACh reduced the number and size of LCRs, and these effects were correlated with the reduction in beating rate. ACh also reduced cAMP levels and the log(concentration)-response curves for reduction in rate and inhibition of phosphorylation of phospholamban were very similar. The authors conclude that there is a tight coupling between suppression of PKA-dependent Ca^2+^ signaling, I_KACh_ activation and reduction of spontaneous rate. van Borren et al. (2010) also investigate the effects of ACh on Ca^2+^ transient, cAMP production and pacemaker frequency. They found that when I(f) was inhibited by 2 mM Cs^+^, and I_KACh_ was inhibited by tertiapin Q, 1 μM ACh was still able to reduce pacemaker frequency by 72%. Under these conditions, there was a good correlation between the reduction in beating rate and the amplitude of the Ca^2+^ transient. In addition ryanodine and exposure to BAPTA-AM both facilitated ACh mediated slowing. They also showed that inhibition of the Ca^2+^ transient by ryanodine (3 μM) or BAPTA-AM (25 μM) exaggerated the ACh-mediated inhibition of cAMP content, consistent with the proposal that Ca^2+^ affects cAMP production in SAN cells. There may be additional effects of ACh on phosphodiesterases (e.g., Han et al., 1995, for a review of PDE subtypes in SAN see Hua et al., 2012) and phosphatases (Ke et al., 2007).

## Summary

The above discussion leads to the view that pacemaker activity in the heart normally requires L-type Ca^2+^ channels and at least one type of voltage-gated potassium channel, while the evidence seems to support the hypothesis that NCX is also essential whether or not it plays a role in determining the poorly understood “background” conductance pathway. The influence of this background conductance pathway allows de-activation of K^+^ channels to cause depolarization toward the threshold for voltage-gated Ca^2+^ channels, and future identification of this pathway will be important for our understanding of pacemaker mechanisms. Pacemaker depolarization is enhanced by activation of I(f). The idea that the I(f) pathway is essential for pacemaker depolarization cannot be completely excluded because of the uncertainties concerning whether the pathway is totally suppressed in experiments using drug blockade or techniques of genetic modification, but it seems likely that I(f) is more an important modulator than an essential component of the timing mechanism. Ca^2+^ sparks/LCRs can sometimes contribute to the later stages of pacemaker depolarization preceding the action potential but are not essential for pacemaking. However, “global” properties of Ca^2+^ uptake and release by the SR across the whole myocyte (or at least a substantial fraction of the SR) can give rise to a “Ca^2+^ clock” (as a consequence of the SR filling with Ca^2+^ to a critical level or from other kinetic properties of the proteins involved in uptake and release of Ca^2+^). The “Ca^2+^ clock” arising in this way frequently plays a modulatory rather than essential role, though there may be conditions in which it is absolutely necessary to re-initiate spontaneous activity if this were to stop. This would mean that Ca^2+^ released from the SR and driving electrogenic NCX may be essential to re-start spontaneous activity under particular conditions. Although a Ca^2+^ clock is not essential for pacemaker activity, it seems probable that under normal conditions there will be a cooperative interaction between membrane and Ca^2+^ clocks that has been referred to as a coupled clock mechanism. Under pathological conditions when the Ca^2+^ clock is not synchronized with the membrane clock the Ca^2+^ clock can lead to disturbed pacemaker rhythms. In normal physiology, Ca^2+^ is thought to drive a depolarizing influence of NCX throughout the cardiac cycle, including during the most negative potential of the pacemaker myocytes, at least under most conditions when the cytosolic Ca^2+^ concentration remains substantially higher than 100 nM. Ca^2+^ also plays important roles in maintaining and regulating pacemaker activity by activating a variety of Ca^2+^-dependent enzymes including CaMKII, AC1, and AC8. Ca^2+^ seems to exert a direct or indirect influence on most if not all of the proteins providing ionic pathways in the surface membrane.

### Conflict of interest statement

The authors declare that the research was conducted in the absence of any commercial or financial relationships that could be construed as a potential conflict of interest.
